# Editorial: The Biofilm Lifestyle of Uropathogens

**DOI:** 10.3389/fcimb.2021.763415

**Published:** 2021-09-17

**Authors:** Veronika Holá, Andrés Opazo-Capurro, Paola Scavone

**Affiliations:** ^1^ Microbiological Institute, Faculty of Medicine of Masaryk University and St. Anne´s University Hospital, Brno, Czechia; ^2^Laboratorio de Investigación en Agentes Antibacterianos (LIAA), Facultad de Ciencias Biológicas, Universidad de Concepción, Concepción, Chile; ^3^Millennium Nucleus for Collaborative Research on Bacterial Resistance (MICROB-R), Santiago de Chile, Chile; ^4^Laboratory of Microbial Biofilms, Department of Microbiology, Instituto de Investigaciones Biológicas Clemente Estable, Montevideo, Uruguay

**Keywords:** biofilm, uropathogens, urinary tract infections, antimicrobials, host response

Biofilms are the common lifestyle of microorganisms in nature and are also involved in many infections. Biofilms are defined as microbial communities irreversibly associated/attached to a biotic or abiotic surface and embedded in an extra-polymeric matrix of their own production. The matrix consists also of several compounds including those from the surrounding environment, e.g. host´s components. Several changes occur during the biofilm formation process, in which genetic, physiological and metabolic differences are observed compared to their planktonic counterparts (Donlan and Costerton, 2002).

It is reported that more than 65% of infections are caused by microorganisms forming biofilms (Lewis, 2001). Urinary tract infections (UTIs) are one of the most common infections in humans; at least 40% of women experience an UTI during their lifetime but also children and older men may suffer from these infections. For many decades, urine and the urinary bladder were considered to be sterile but this dogma has been changed as it was demonstrated that there are many microorganisms living with us in our bladders (Thomas-White et al., 2016). However, the relevance and the contribution of these microorganisms in health and disease remains under investigation. In the case of UTIs, the etiological agents are comprised mainly of *Escherichia coli*, *Klebsiella pneumoniae*, *Proteus mirabilis*, *Enterococcus faecalis* and *Staphylococcus saprophyticus*, but other microorganisms are emerging as UTI agents, such as *Acinetobacter baumannii* or *Candida albicans*. Most of these uropathogens can form biofilms, where this capability could be related to the recurrence and persistence of this infection. This strategy can help microorganisms to survive in such a stressful environment with low nutrient availability and an active immune system. Antimicrobial resistance is really high among the uropathogens but not only intrinsic resistance is observed, such biofilm formation is also reported as a mechanism of resistance (Jamal et al., 2018).

In this Research Topic there are different contributions that aim to understand the role of biofilms produced by uropathogens and other microorganisms causing UTIs, catheter associated UTIs, polymicrobial biofilm infections, the host response to biofilms, and biofilm s as a virulence factor in the human urinary tract.

The most common uropathogen is the uropathogenic *Escherichia coli* (UPEC) which cause more than 75% of the UTIs (Flores-Mireles et al., 2015). One strategy developed by UPEC consists of forming biofilm-like intracellular bacterial communities (IBCs) that protect bacteria from neutrophils, antibiotics and it is related to the high recurrence of *E. coli* UTI infection. González et al. demonstrated that clinical UPEC isolates were able to invade a bladder cell line. Moreover, they find that three antibiotics (ceftriaxone, ciprofloxacin and azithromycin) significantly decreased the number of intracellular bacteria. These *in vitro* findings could improve the treatment of UPEC infections and its high recurrence. Other research performed by Vasudevan et al. evaluated the use of phytomolecules (Type A procyanidin) with nitrofurantoin against a multi-drug resistant biofilm forming UPEC strain. The authors concluded that synergy was observed with the phytomolecule and the antibiotic and also an anti-biofilm activity was reported. Treatment of UTIs is a big challenge in which nanotechnology appears as a novel strategy. In this context, Sanchez et al. reviewed the literature in this area and described different approaches with a potential impact in UTI treatment. Different types of nanoparticles (NP), such as organic, nanodiamonds, chemical and green inorganic NP could have different potential applicability.

In addition to UPEC, another relevant uropathogen is *P. mirabilis.* It is well know that it produce biofilms and it is responsible for catheter associated urinary tract infections (CAUTI). *P. mirabilis* also produces urease that hydrolyses urea with the concomitant production of carbon dioxide and ammonium. Consequently, the urine pH increases which leads to the production of calcium crystals and magnesium ammonium phosphate precipitates. *P. mirabilis* biofilms are crystalline and it is a problem in catheterized patients as it blocks the catheter with serious consequences to the patients. In this context, Wasfi et al. reviewed the state-of-the-art of the mechanisms by which *P. mirabilis* forms biofilms and how we can prevent biofilm formation using natural and synthetic compounds, including the use of bacteriophages. Also, Czerwonka et al. identified the genes involved in LPS decoration in *P. mirabilis*. The authors sequenced the genome of a *P. mirabilis* serogoup O18 strain. Several features are interesting in this strain concerning the organization of the O antigen gene cluster. Moreover, the authors compared the complete genome with other *P. mirabilis* genomes available.

Even though *E. coli* and *P. mirabilis* are the most common uropathogens, *A. baumannii* has been recently recovered from urinary samples and, thus, is being recognized as an important uropathogen. Colquhoun and Rather reviewed the latest findings in *A. baumannii* regarding biofilm formation, urinary tract colonization and pathogenesis.

This Research Topic clearly contributes to the understanding of the biofilm lifestyle of uropathogens and presents state-of-the-art information about this relevant research field, which represents an important challenge that should be addressed.

## Author Contributions

PS wrote the first draft of the manuscript. VH and AC corrected the first draft. All authors contributed to the article and approved the submitted version.

## Conflict of Interest

The authors declare that the research was conducted in the absence of any commercial or financial relationships that could be construed as a potential conflict of interest.

## Publisher’s Note

All claims expressed in this article are solely those of the authors and do not necessarily represent those of their affiliated organizations, or those of the publisher, the editors and the reviewers. Any product that may be evaluated in this article, or claim that may be made by its manufacturer, is not guaranteed or endorsed by the publisher.
